# Validity of three smartwatches in estimating energy expenditure during outdoor walking and running

**DOI:** 10.3389/fphys.2022.995575

**Published:** 2022-09-26

**Authors:** Shenglong Le, Xiuqiang Wang, Tao Zhang, Si Man Lei, Sulin Cheng, Wu Yao, Moritz Schumann

**Affiliations:** ^1^ Exercise Translational Medicine Center, Shanghai Center for Systems Biomedicine, Shanghai Jiao Tong University, Shanghai, China; ^2^ Department of Physical Therapy, Taihe Hospital, Hubei University of Medicine, Shiyan, China; ^3^ Key Laboratory of Systems Biomedicine (Ministry of Education), Shanghai Center for Systems Biomedicine, Shanghai Jiao Tong University, Shanghai, China; ^4^ Faculty of Sport and Health Sciences, University of Jyväskylä, Jyväskylä, Finland; ^5^ Faculty of Education, University of Macao, Macao, China; ^6^ Physical Education Department, Shanghai Jiao Tong University, Shanghai, China; ^7^ Department of Molecular and Cellular Sport Medicine, German Sport University, Cologne, Germany

**Keywords:** wearable devices, validation, accuracy, physical activity, health monitoring

## Abstract

Commercially wrist-worn devices often present inaccurate estimations of energy expenditure (EE), with large between-device differences. We aimed to assess the validity of the Apple Watch Series 6 (AW), Garmin FENIX 6 (GF) and Huawei Watch GT 2e (HW) in estimating EE during outdoor walking and running. Twenty young normal-weight Chinese adults concurrently wore three index devices randomly positioned at both wrists during walking at 6 km/h and running at 10 km/h for 2 km on a 400- meter track. As a criterion, EE was assessed by indirect calorimetry (COSMED K5). For walking, EE from AW and GF was significantly higher than that obtained by the K5 (*p* < 0.001 and 0.002, respectively), but not for HW (*p* = 0.491). The mean absolute percentage error (MAPE) was 19.8% for AW, 32.0% for GF, and 9.9% for HW, respectively. The limits of agreement (LoA) were 44.1, 150.1 and 48.6 kcal for AW, GF, and HW respectively. The intraclass correlation coefficient (ICC) was 0.821, 0.216 and 0.760 for AW, GF, and HW, respectively. For running, EE from AW and GF were significantly higher than the K5 (*p* < 0.001 and 0.001, respectively), but not for HW (*p* = 0.946). The MAPE was 24.4%, 21.8% and 11.9% for AW, GF and HW, respectively. LoA were 62.8, 89.4 and 65.6 kcal for AW, GF and HW, respectively. The ICC was 0.741, 0.594, and 0.698 for AW, GF and HW, respectively. The results indicate that the tested smartwatches show a moderate validity in EE estimations for outdoor walking and running.

## Introduction

Wearable technology has been ranked as the top fitness trend for several years (Thompson, 2021) and in 2021 the worldwide shipments of wearable devices reached 533.6 million units (Laricchia, 2022). Among these, wrist-worn devices are most common and are capable of monitoring a large variety of vital parameters including steps taken, distance travelled, heart rate and energy expenditure (EE) with a variety of sensors. Given their convenience, the data provided by wearable devices are often used to monitor and/or modify health behaviors both for self-monitoring as well as in healthcare and research settings (Lyons et al., 2014). The use of the devices is typically encouraged by marketing claims set out by the manufacturers, while actual data on the validity of the devices is typically lacking (Evenson et al., 2015).

Estimating EE by wrist-worn wearable devices seems convenient, considering the linear relationship of heart rate and/or physical activity with gaseous exchange. Consequently, numerous validation studies on EE estimations have been conducted, but provided heterogeneous results (O’driscoll et al., 2020; Argent et al., 2022). Overall, it appears that commercially available wrist- or arm-worn devices did not show a sufficient accuracy, while large between-device differences were observed (O’driscoll et al., 2020).

The differences in the accuracy of different devices may be dependent on several factors. Most importantly, the accuracy of the input parameters, such as the accuracy of heart rate from photoplethysmography (PPG) sensors affects EE estimations (O’driscoll et al., 2020; Argent et al., 2022). In this context, it appears that the accuracy of EE estimation may vary with the type and intensity of activities (O’driscoll et al., 2020). While this may be related to motion affecting the accuracy of the PPG signal, the observed error for different types of activities may also be related to the algorithms that likely do not take physical activity type or bodily posture into account (Schneller et al., 2015). Several studies found that algorithm adjustments may indeed improve the validity of the EE estimation during exercise (Jakicic et al., 2004; Van Hoye et al., 2015). Furthermore, addition of heart rate or heat sensors to accelerometer can improve the accuracy of EE estimations compared to accelerometry alone (O’driscoll et al., 2020; Kinnunen et al., 2019). Collectively, it appears that further technological advancements and revised algorithms may improve the quality of EE estimations. Thus, continuous validations of devices that are newly introduced to the market are indispensable.

For the thorough validation of EE estimations, a number of factors should be considered. Among the most important variables appears to be the intended use of the device, i.e. validating EE estimations in settings that represent the actual use by consumers (Argent et al., 2022). This is because specific sensors may be used for specific activities. In this regard, outdoor activities may be supported by GPS signal while indoor activities may solely rely on accelerometry, likely compromising the accuracy (Charlot et al., 2014). Therefore, this study aims to examine the accuracy of the EE estimations of three new generations of smartwatches of the leading manufacturers, namely the Apple Watch Series 6 (AW), Garmin Fenix 6 (GF) and Huawei GT 2e (HW) during outdoor walking and running in young Chinese adults.

## Materials and methods

### Participants

Twenty healthy Chinese subjects (10 males and 10 females) were recruited from the local university campus (Table 1). On the bases of the paired sample *t*-test, a post-hoc analysis was performed to estimate if sample size is sufficient with proper effect size from three devices under both walking and running conditions by G*Power version 3.1.9.6 (Franz Faul, University Kiel, Germany) using the mean and standard deviations between criterion device (K5) and test device (Passler et al., 2019). For AW, GF and HW, the statistical power is 1.000, 0.929 and 0.105 for the outdoor walking, respectively, while 0.999, 0.942 and 0.051 for the outdoor running respectively. Participants were screened for inclusion criteria using the lifestyle and disease questionnaire and the ACSM Medical History, Signs and Symptoms, and Risk Factors for Risk Stratification (Jonas, 2009). Inclusion criteria included healthy young adults (aged 18–30 years) with a normal weight (body mass index 18.5–25 kg/m^2^) and self-reported regular recreational exercise (≥3 weekly exercise sessions). All participants were informed about the study procedures and provided written informed consent prior to the testing. The study was conducted in accordance with the declaration of Helsinki and approved by the Institional Review Board for Human Research Protections of Shanghai Jiao Tong University (registration number B2020024I). Criterion measure assessment.

**TABLE 1:** Physical characteristics of participants.

	Male (n = 10)	Female (n = 10)	All participants (n = 20)
Age (yr)	23.8 ± 1.2	22.1 ± 2.7	23.0 ± 2.2
Height (cm)	178.5 ± 8.3	168.7 ± 8.5	173.6 ± 9.6
Weight (kg)	71.7 ± 10.3	62.6 ± 10.3	67.1 ± 11.1
BMI (kg/m2)	22.4 ± 2.0	21.8 ± 1.9	22.1 ± 1.9
BF (%)	14.5 ± 4.8	23.8 ± 5.6	19.2 ± 6.9

Data are expressed as mean ± SD. BMI, body mass index; BF, body fat percentage; SD, standard deviation.

As the criterion, the Cosmed K5 (K5) system (Cosmed, Italy) was used. K5 is a portable gas analysis system using Breath-by-Breath technology. This technology enables a precise and accurate determination of the individual VE, VO2 and VCO2 for a wide range of metabolic rates (Deblois et al., 2021). The estimation of EE is based on the ratio of inhaled oxygen to exhaled carbon dioxide. Respiratory gases of each breath are analyzed. Before each measurement, the device was warmed up for a minimum of 15 min and calibrated with both high-grade calibration gases and a 3 L calibration syringe according to the recommendations by the manufacturer. Mask size was individually fitted prior to the test and maintained throughout the entire measurement.

### Index device assessment

This study examined the validity of the Apple Watch Series 6 (AW), Garmin Fenix 6 (GF), and Huawei GT 2e (HW), all of which were the newest generations introduced in January 2021. Each smartwatch has the outdoor walking and running mode, and records distance, speed and heart rate in real time. All devices use photoplethysmography to estimate heart rate from the wrist and use the GPS to estimate distance and speed during the outdoor walking and running. EE was estimated in real time and displayed.

All watches used in the study were bought commercially. Three watches were placed two on one wrist and one on the other wrist for different participants according to a pre-randomly allocation list in order to keep the number of devices on one wrist counterbalanced. The position of the watch was selected as careful as possible according to the manufacturer’s instructions.

### Protocol

Data for this study were collected during one visit. Subjects were instructed not to consume food, coffee, tea, or other stimulants e.g. energy drinks or soft drinks except water at least 6 h prior to measurements and not to do any vigorous physical activity and consume alcohol during 24 h prior to measurements. Subjects’ height was measured to the nearest 0.1 cm using a wall-mounted height scale. Body mass and body composition were measured after emptying the bladder and in light underwear using a calibrated InBody 720 bio-impedance device (Biospace, Co, Ltd., Seoul, Korea). Height, weight, gender, and date of birth were used to initialize the smartwatches for each subject.

**FIGURE 1:**
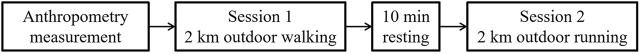
Experimental protocol. In the first session, the subjects were advised to walk a 2-km distance at approximately 6 km/h. In the second session, the subjects were asked to run a 2-km distance at approximately 10 km/h. Two sessions were separated by 10 minutes sitting break.

After the anthropometry measurement, the protocol included two exercise sessions performed on the outdoor running track as showed in Figure 1. Two sessions were separated by a recovery period, which was 10 minutes sitting break. In the first session, the subjects were advised to walk a 2-km distance at approximately 6 km/h. In the second session, the subjects were asked to run a 2-km distance at approximately 10 km/h. Subjects were instructed to keep a steady pace during the session. The walking speed was 6.15 ± 0.29 km/h and the running speed was 10.94 ± 0.99 km/h. The examiner confirmed that heart rate had returned to resting levels before the second session. The corresponding outdoor walking or running mode was selected and started and ended at the same time for all smartwatches. The starting and end time of each session was recorded on the data sheet by researchers. The temperature and relative humility was 16.5 ± 2.6°C.and 56.7 ± 14.3% for walking and 13.9 ± 2.9°C.and 62.6 ± 21.8% for running.

### Data acquisition and processing

The criterion data from the K5 were downloaded breath-by-breath and included measures of EE. EE values from the K5 were summed individually for each exercise session. The EE estimates from the watches were obtained directly from screenshots of the respective applications, because the watches primarily reported cumulative totals. Due to technical errors, we only obtained 19 EE data from HW for walking and running, respectively.

### Data analyses

Statistical analyses were conducted using IBM SPSS Statistics software version 24 (IBM, Armonk, New York). Descriptive statistics were used to characterize the sample population. The validity of the watches was determined by several statistical tests. Data from the smartwatches were compared with the criterion using paired sample t-tests. The mean absolute percentage errors (MAPE) were calculated as an indicator of measurement error. The absolute percentage errors (%) were calculated as follows: |EE from smartwatches - EE from K5|/(EE from K5) ×100 for each subject. A MAPE of ≤10% was used as the criterion value for validity (Nelson et al., 2016). As the commonly used method to validate wearable devices, the Intraclass Correlation Coefficient (ICC) defined the agreement between the gold standard and the tested devices, providing an estimate of overall concordance between two methods (Fokkema et al., 2017; Wahl et al., 2017; Boudreaux et al., 2018). Excellent, good, moderate, and low agreement thresholds were defined as ICC values of ont, goo). Excellent, gooices, providin (Fokkema et al., 2017). To investigate the level of agreement, Bland-Altman plots were prepared according to Bland and Altman (Bland and Altman, 1986). For this, limits of agreement were set to 95%.

## Results

### EE during the outdoor walking

The EE during the outdoor walking was showed in Table 2. On average, participants achieved an EE of 108.7 ± 17.4 kcal in K5 during the outdoor walking. The average estimated EE was 129.1 ± 20.1 kcal, 139.6 ± 39.6 kcal and 111.2 ± 18.1 kcal from AW, GF and HW, respectively. Paired *t*-test analysis showed that EE from AW and GF were significantly higher than that obtained by the K5 (*p* < 0.001 and 0.002, respectively), but not significantly different for HW (*p* = 0.491). Compared to the K5, the MAPE of EE was 19.8%, 32.0% and 9.9% in AW, GF and HW, respectively (Table 2). Individual EE values from AW (ICC = 0.821) and HW (ICC = 0.760) also showed good agreement with K5. However, the agreement for GF (ICC = 0.216) was low (Fokkema et al., 2017).

**TABLE 2:** Descriptive examination of the differences between the estimated EE (smartwatches) and the measured EE (K5) during the outdoor walking and running.

Activity	Device	N	EE (kcal)	Diff (kcal)	MAPE (%)	ICC	t	*p*	*p*
	AW	20	129.1 ± 20.1	20.5 ± 11.3	19.8 ± 12.4	0.821	−8.129	0.000	1.000
Walking	GF	20	139.6 ± 39.6	31.0 ± 38.3	32.0 ± 34.1	0.216	−3.615	0.002	0.929
	HW	19	111.2 ± 18.1	2.0 ± 12.4	9.9 ± 8.2	0.760	−0.703	0.491	0.105
	K5	20	108.7 ± 17.4	NA	NA	NA	NA	NA	NA
	AW	20	137.8 ± 23.1	25.7 ± 16.0	24.4 ± 16.1	0.741	−7.162	0.000	0.999
Running	GF	20	137.8 ± 23.1	19 ± 22.8	21.8 ± 17.3	0.594	−3.715	0.001	0.942
	HW	19	111.8 ± 21.1	−0.3 ± 16.7	11.9 ± 9.9	0.698	0.069	0.946	0.051
	K5	20	112.2 ± 21.3	NA	NA	NA	NA	NA	NA

Data are expressed as mean ± SD, for EE, Diff and MAPE., Data from the smartwatches were compared with the criterion using paired sample t-tests. EE, energy expenditure; Diff: the difference of the estimated EE (smartwatches) with the measured EE (K5); K5, the golden standard of EE, assessment; AW, Apple Watch Series 6; GF, Garmin FENIX, 6; HW, Huawei Watch GT, 2e; MAPE, mean absolute percentage error; ICC, intraclass correlation coefficient; SD, standard deviation; *p*, statistical power.

**FIGURE 2:**
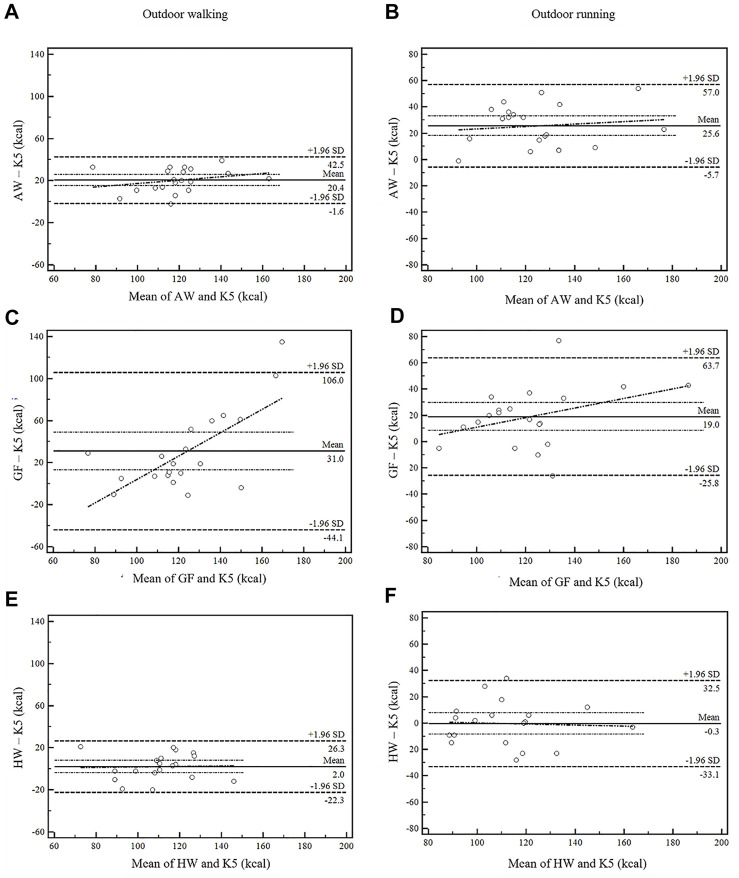
Bland-Altman plots comparing the EE estimations by smartwatches (AW **(A,B)**, GF **(C,D)**, HW **(E,F)**) and K5. The differences of the EE values on the *y*-axis relative to the mean of the two methods (smartwatch and K5) on the *x*-axis. Mean differences (bias) between estimated EE and EE of criterion, upper and lower limits of agreement (ULoA, LLoA) are labeled in the plots. Limits of agreement (LoA) were calculated as means ±1.96 SD. K5, the criterion of EE assessment; AW, Apple Watch Series 6; GF, Garmin FENIX 6; HW, Huawei Watch GT 2e; SD, standard deviation.

In the Bland-Altman plots, the percentage of the values within the limits of agreement was 100%, 90% and 95% for AW (Figure 2A), GF (Figure 2C) and HW (Figure 2E), respectively. The AW showed the narrowest limits of agreement (44.1 kcal), followed by HW of 48.6 kcal, while the GF exhibited the broadest limits of agreement (150.1 kcal).

### EE during the outdoor running

The EE during the outdoor running was showed in Table 2. On average, participants achieved an EE of 112.2 ± 21.3 kcal in K5 during the outdoor running. The average estimated EE was 137.8 ± 23.1 kcal, 131.1 ± 28.8 kcal and 111.8 ± 21.1 kcal from AW, GF and HW, respectively. Paired *t*-test analysis showed that EE from AW and GF were significantly higher than the K5 (*p* < 0.001 and 0.001, respectively), but not significantly different for HW (*p* = 0.946). Compared to the K5, the MAPE of EE was 24.4%, 21.8% and 11.9% in AW, GF and HW, respectively. Individual EE values from AW (ICC = 0.741) and HW (ICC = 0.698) also showed moderate agreement with K5. However, the agreement for GF (ICC = 0.594) was low (Fokkema et al., 2017).

In the Bland-Altman plots, the percentage of the values within the limits of agreement was 95% for AW (Figure 2B) and GF (Figure 2D) and 100% for HW (Figure 2F). The AW showed the narrowest limits of agreement (62.8 kcal), followed by HW of 65.6 kcal, whereas the GF exhibited the broadest limits of agreement (89.4 kcal).

## Discussion

This study investigated the validity of three popular smartwatches (AW, GF, HW) for estimating EE during outdoor walking and running by means of a portable gas analysis system (K5) as the criterion. The results revealed that some, but not all, of the smart-watches provide a reasonably accuracy for EE estimation. Estimations of EE provided by AW and HW demonstrated good/moderate criterion agreement, while the EE from GF showed poor agreement.

Although the HW yielded the best overall results, the AW also performed well in this study. The HW showed the lowest MAPE (9.9% for walking and 11.9% for running), while AW had the narrowest limits of agreement (44.1 kcal for walking and 62.8 kcal for running). Indeed, a MAPE of <10% can be considered as a high accuracy (Nelson et al., 2016). Since the manufacturer of HW is based in China and a Chinese population was included in this study it is likely that this explains the high accuracy. In a previous study it was shown that the accuracy of EE estimations by activity monitors differed between different ethnicities (Brazeau et al., 2014), but in that study only accelerometer data was used for EE estimations. Unfortunately, most manufacturers do not provide any details on the algorithms that are used for EE estimations and as such, it remains unknown which variables were used in the devices tested in the present study. However, it is likely that a combination of accelerometry, PPG and GPS derived data was utilized. As such, we are only able to assess the ecological validity that is the result of both sensor quality and algorithm but the actual source of inaccuracy especially observed in GF remains unknown.

There is considerable variability of device accuracy for predicting EE of different types of activities (O’driscoll et al., 2020; Lee et al., 2014). The present study demonstrated that overall error estimates were similar between walking and running for AW and HW, but different for GF. GF could likely provide better EE estimates for the outdoor walking (MAPE = 32.0%) than running (MAPE = 21.8%). Wahl et al. (Wahl et al., 2017) examined the influence of running pace on the EE. The study shows a significant influence of running pace on the estimated EE. Energy expenditure tends to be overestimated at lower pace and underestimated at higher pace. Roos et al. (Roos et al., 2017) found that sportswatches significantly underestimated EE during the high intensity running with a proportional error increasing as the running speed increased. Both studies suggested improving the EE estimation algorithm in all range speed (Roos et al., 2017; Wahl et al., 2017). Nevertheless, this diversity also may be related to inaccuracies of the sensor (e.g. movement artefacts) at different speed. These observations indicated that consumer/health professionals should take more attention to select the most accurate device for the distinct types of activity.

Smartwatch EE overestimated EE versus the K5 during the outdoor walking and running. These observations are congruent with previous studies (Bai et al., 2016; Pope et al., 2019). EE during outdoor walking was overestimated by 19.8 % and 32.0% by the AW and GF, respectively, while the energy cost of running was overestimated by 24.4 % and 21.8%. Considering smartwatch EE data may be used for the purpose of maintaining or even losing body weight, these observations are remarkable. For example, the individual may set the goal of increasing the EE by 250 kcal per day through walking or running in order to lose 1 kg body weight per month. However, due to the smartwatch’s EE data inaccuracy, the actual increasing daily EE may be less than 200 kcal, resulting in the ineffectiveness of the weight loss program. Our findings, therefore, suggest a cautious approach to be taken when interpreting smartwatch EE data given the observed EE overestimation.

When interpreting our findings, one needs to be bear in mind a few limitations. Most notably, our study was designed to include only outdoor walking and running with advised self-selected steady speed. Furthermore, our findings may not translate directly into other activities due to e.g. differences in motion artefacts. In addition, our sample was restricted to healthy young Chinese within the normal range of BMI. Therefore, we cannot generalize these findings to the other diverse population groups, such as elderly or obese. Furthermore, as the body side was randomized, we side specific effects of the accuracy. Future studies should consider powering the study to compare the effects of different wearing locations on the accuracy of the devices. Finally, this study did not assess the reliability of EE estimation from the smartwatches. Despite issues with accuracy, the reliability is very important for individuals and health professionals to monitor changes in EE over time. Future studies should incorporate reliability testing as well.

## Conclusion

The findings of this study indicate that the smartwatches of AW, GF and HW may generally have moderate validity in EE estimates for outdoor walking and running. Small sample size with large variables even had sufficient power still a limit factor to generalize the results for different populations. Consumers should also be cautious when using the tested smart-watches for prediction of their energy expenditure.

## Data Availability

The raw data supporting the conclusions of this article will be made available by the authors, without undue reservation.
